# Octopus-Inspired Modular Two-Segment Pneumatic Soft Manipulator with Passive Suction Cups

**DOI:** 10.3390/biomimetics11080558

**Published:** 2026-08-05

**Authors:** Siyu Mei, Tongtong Ma, Rensong Yin, Chong Liu, Hui Chen

**Affiliations:** 1 Center for Mechanics Plus Under Extreme Environments, School of Mechanics and Engineering Science, Ningbo University, Ningbo 315211, China; 2411090194@nbu.edu.cn (S.M.); 2311090160@nbu.edu.cn (T.M.); 2211090042@nbu.edu.cn (R.Y.); 2401090039@nbu.edu.cn (C.L.); 2 Piezoelectric Device Laboratory, School of Mechanics and Engineering Science, Ningbo University, Ningbo 315211, China

**Keywords:** bio-inspired soft manipulator, modular pneumatic actuator, two-segment continuum arm, passive suction cup, piecewise constant curvature

## Abstract

Octopus arms combine a compliant continuum body with distributed suckers, providing a biological reference for soft manipulators that require large deformation and stable local contact. Inspired by this functional organization, this study presents an octopus-inspired two-segment pneumatic soft manipulator with passive suction cups at the distal end. The manipulator consists of a cylindrical proximal segment, a tapered distal segment, and a thermoplastic polyurethane (TPU) suction-cup array. The proximal segment provides structural support and global bending, whereas the tapered distal segment improves local compliance and contact posture adjustment near the target surface. Each segment contains three independently driven pneumatic chambers arranged at 120° intervals, enabling spatial bending through differential pressurization. The distal suction cups are not connected to an active vacuum source; instead, attachment is assisted by mechanical pressing, partial air expulsion from the cup cavity, and elastic recovery of the cup lip. Finite element simulations were conducted to examine pressure-driven bending of the soft arm and deformation of the suction cups under equivalent sealing loads. A piecewise constant curvature model was established to estimate the posture and reachable workspace of the two-segment manipulator. A prototype was fabricated and tested on a pneumatic control platform. Within the pressure range of 50–200 kPa, both segments exhibited increasing bending angles with increasing input pressure; at 200 kPa, the maximum observed bending angles were approximately 70° for the proximal segment and 87° for the distal segment. Distal-segment tests demonstrated passive contact holding on a brown glass bottle and a black roll of electrical tape. Coordinated actuation further produced compound bending and twisting postures. These results show that the proposed design translates the functional division of octopus arms into a modular pneumatic soft manipulator with controllable spatial deformation and passive distal contact support.

## 1. Introduction

Robotic arms operating in cluttered, narrow, or contact-rich environments must be able to reach target objects while tolerating unintended contact and adapting to uncertain geometries. Conventional rigid manipulators provide high accuracy and load capacity, but their discrete links and joints limit their compliance when interacting with irregular objects or confined spaces [1,2,3]. Soft robots offer a different solution by using body deformation as part of the interaction process [4,5,6]. Among different actuation methods, pneumatic actuation is widely used in soft manipulators because it enables lightweight structures, large deformation, and intrinsic compliance [7,8,9,10]. By arranging multiple chambers around a soft body, a pneumatic segment can bend in different directions or extend under pressure [11,12,13,14].

For manipulation tasks, however, deformation alone does not always ensure stable interaction. A soft arm may conform to an object, but slipping can still occur when the contact area is limited, the surface is smooth, or the object cannot be fully enclosed. Existing studies have improved contact stability using shape-adaptive grippers, variable-stiffness structures, and suction-assisted soft end effectors [15]. In parallel, modeling and design methods for soft continuum robots have been developed using curvature-based models, fabric-based actuators, helical pneumatic actuators, rod-based pneu-net models, inverse design, and lattice-reinforced pneumatic structures [16,17,18,19,20,21]. These studies provide useful foundations for soft manipulator design, but the distal contact interface remains an important factor affecting manipulation stability [22,23]. Octopus arms provide a useful biological reference for this problem. An octopus arm does not rely on rigid skeletal support or a single terminal gripper. Instead, the compliant arm body provides continuous bending and reaching, while distributed suckers provide local and reversible attachment to surrounding surfaces [24,25,26]. Recent work has explored vacuum-powered soft bending actuators with programmable curvature [27], octopus-inspired arms with suction cups [28], tapered soft actuators with suckers [29], bio-inspired attachment mechanisms [30], and dual-segment soft arms with integrated cups [31]. Deformation-adaptive sensing arrays further indicate a possible route toward state monitoring in compliant robotic systems [32]. This biological organization suggests a practical design principle: the continuum body can provide reaching and posture adjustment, while the distal suction interface can assist local contact after the arm reaches the target. Table 1 compares the present design with closely related octopus-inspired systems. The distinguishing feature of the present design is the integration of separately actuated cylindrical and tapered pneumatic segments with a lightweight, passive, vacuum-free TPU contact array.

Based on this principle, this study develops an octopus-inspired modular two-segment pneumatic soft manipulator with passive suction cups at the distal end. The manipulator consists of a cylindrical proximal segment, a tapered distal segment, and a TPU suction-cup array. The proximal segment is designed for structural support and global bending, whereas the tapered distal segment improves local compliance and contact posture adjustment near the object. The suction cups are not connected to an active vacuum source; they provide passive attachment through mechanical pressing and elastic recovery of the cup lip. In this way, the proposed design follows the functional division of octopus arms, combining a deformable pneumatic body for motion generation with a lightweight distal interface for local contact support. The main contributions of this work are as follows. First, the functional division of octopus arms is translated into a modular two-segment pneumatic architecture, in which the proximal and distal segments can be actuated separately to generate compound spatial postures. Second, finite element simulation, piecewise constant curvature modeling, and workspace analysis are used to evaluate the pressure-driven deformation and reachable region of the manipulator. Third, prototype experiments are conducted to verify the pressure–bending response, passive suction-cup-assisted distal contact, and coordinated two-segment motion.

## 2. Biomimetic Design and Fabrication

### 2.1. Biological Inspiration and Design Principle

**Figure 1:**
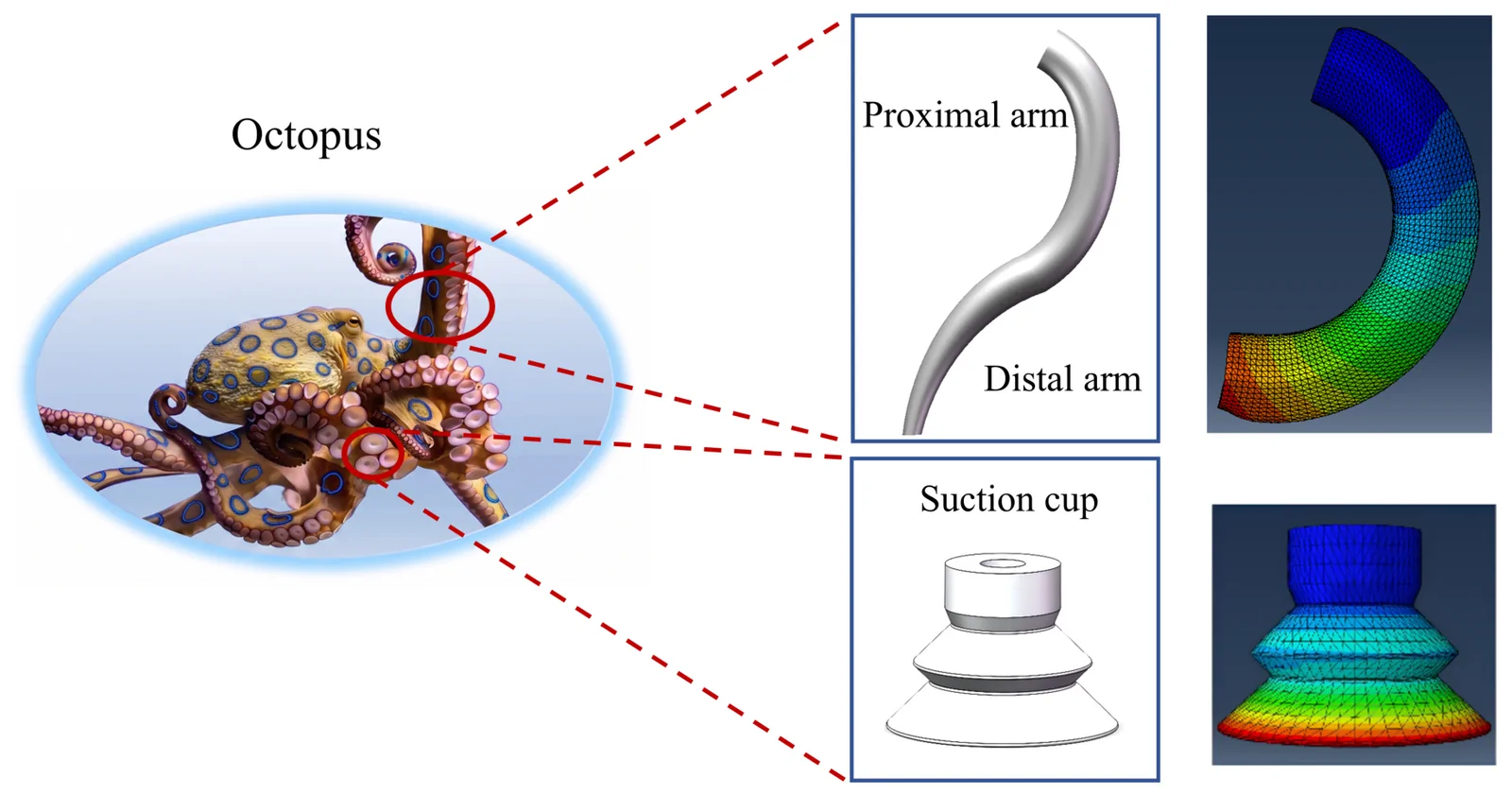
Biological inspiration from octopus arms and the corresponding bio-inspired design concept of the soft manipulator.

An octopus arm combines two functions that are usually separated in conventional robots. Its muscular body changes shape continuously, while its suckers provide local and reversible attachment to surrounding surfaces. The body therefore gives the animal reach and compliance, and the suckers help maintain contact after the arm reaches an object or substrate. This functional division was used as the design principle of the proposed manipulator. The pneumatic continuum arm is responsible for reaching and bending, while the distal suction-cup array provides local contact support after mechanical pressing, as shown in Figure 1.

### 2.2. Manipulator Structure

**Figure 2:**
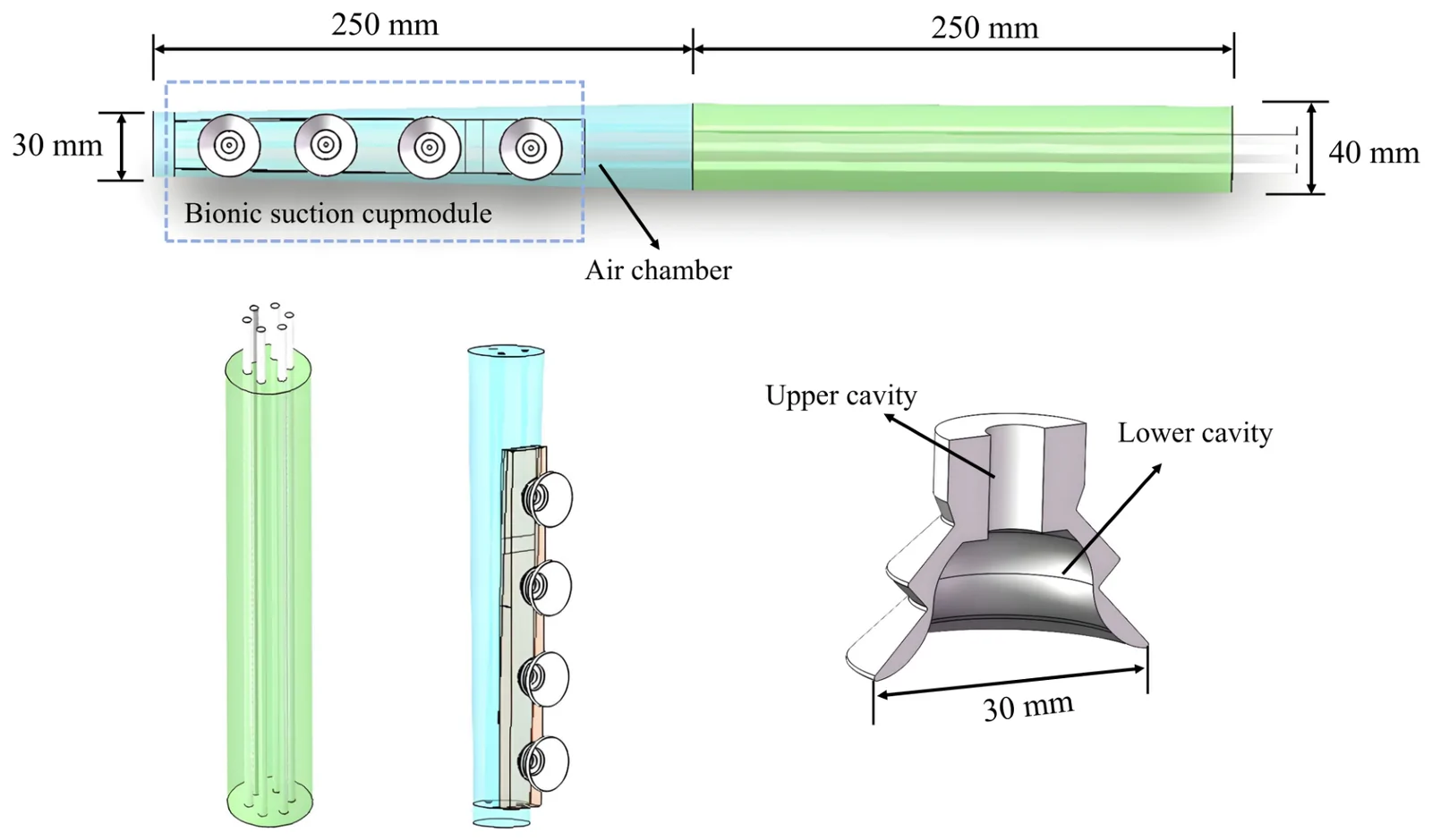
CAD structural design and schematic representation of the dual-segment pneumatic soft manipulator integrated with bio-inspired suction cups.

The manipulator consists of two serial pneumatic segments and a suction-cup array mounted at the distal end (Figure 2). The total length is approximately 500 mm, with each segment about 250 mm long. The proximal segment has a cylindrical silicone body with a diameter of 40 mm. The distal segment tapers from 40 mm near the joint to 30 mm near the tip. The taper ratio, segment lengths, chamber dimensions, and cup arrangement were selected as engineering parameters constrained by chamber layout, mold casting, TPU printing, tube connection, and assembly; they were not obtained from quantitative anatomical parameterization or global optimization. The two segments are connected in series to provide a continuous soft body, while still allowing the proximal and distal sections to perform different mechanical roles during bending and contact. Both segments are made of silicone rubber with a Shore hardness of 40A. The proximal segment contains six circumferential channels. Three channels serve as actuation chambers arranged at 120° intervals, and the remaining channels are used to route pressure to the distal segment. The distal segment also contains three independent actuation chambers arranged at 120° intervals. The proximal and distal actuation chambers are supplied through separate pneumatic paths and do not form one shared internal cavity. Pressurizing one chamber bends the segment toward the opposite side, and changing the pressurized chamber changes the bending plane. This modular arrangement allows the proximal and distal segments to be driven separately and to form compound spatial postures.

The suction-cup array is printed from TPU and contains four cups with a diameter of 30 mm. The cup-wall thickness is 2.5 mm, and the cups are fabricated from 85A polyether TPU without an embedded rigid supporting structure. Flexible silicone links connect the cups so that the distal interface can adapt to mildly uneven surfaces. This linked arrangement also allows the cups to share local deformation during contact, rather than behaving as a rigid end effector. The array therefore works as a compliant contact interface between the soft arm and the target surface. In the present prototype, the cups are not connected to an active vacuum source. During contact, the arm presses the cups against the target surface, part of the enclosed air is expelled, and elastic recovery of the cup lip helps maintain a local seal. No controlled pull-off-force, holding-duration, or repeated-cycle test was performed; the contact demonstrations are therefore interpreted qualitatively. This passive design keeps the distal end light and compliant, although the holding force depends on surface condition, local curvature, pressing force, and cup-lip deformation.

### 2.3. Finite Element Simulation

Finite element analysis was used before prototype testing to check the arm deformation and the suction-cup response. Finite element models were developed in Abaqus to examine pressure-driven arm deformation and the structural response of the suction cups. The material models, mesh settings, boundary conditions, and loading cases are summarized in Table 2.

**Figure 3:**
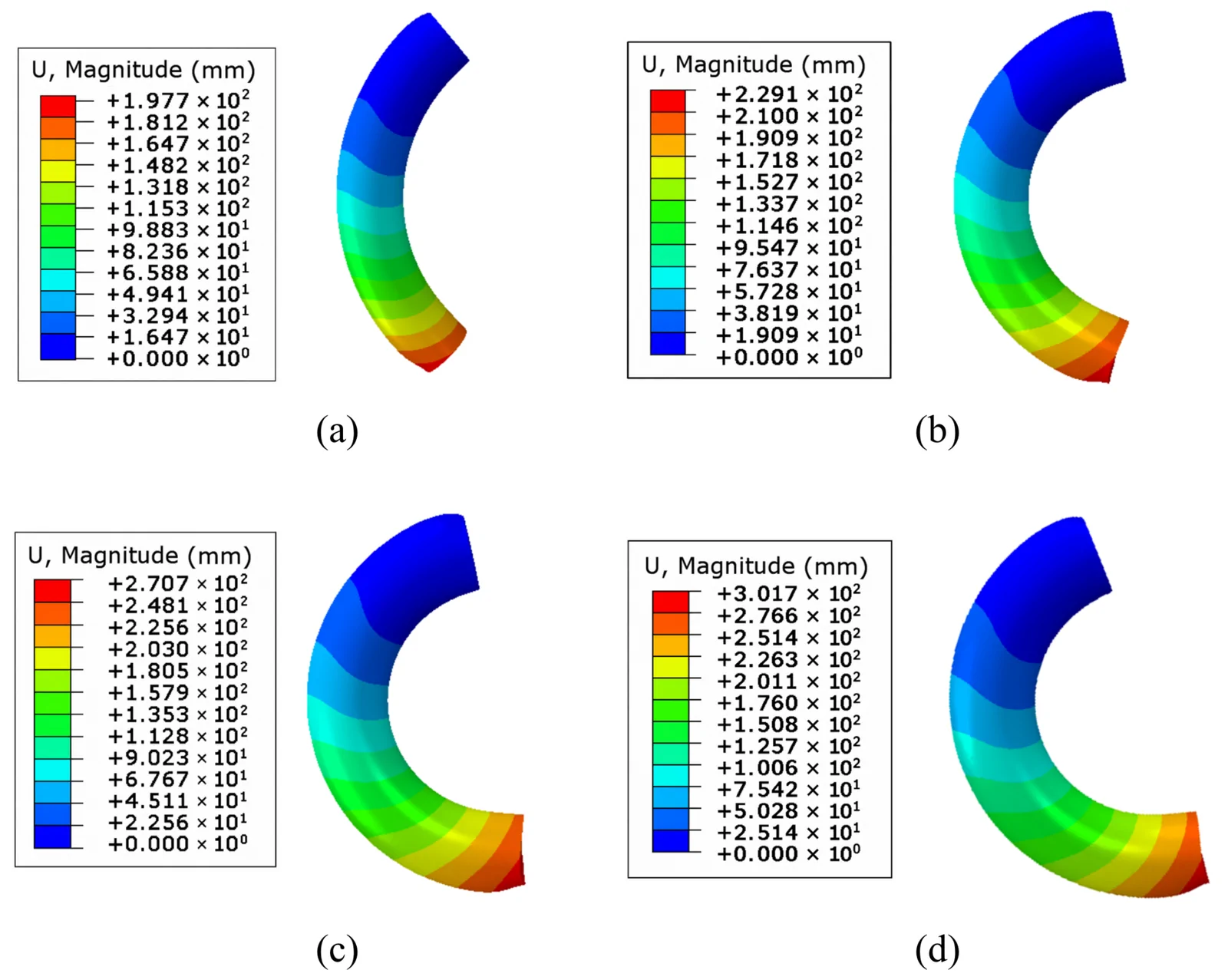
Finite element simulation results of soft-manipulator bending under different driving pressures: (**a**) 50 kPa; (**b**) 100 kPa; (**c**) 150 kPa; (**d**) 200 kPa.

For the arm model, one proximal chamber was pressurized at 50, 100, 150, and 200 kPa, respectively, and the resulting displacement and von Mises stress were evaluated. Each load case was applied to the same chamber wall, while the remaining two proximal chambers were maintained at zero gauge pressure. This configuration isolates the bending response of one proximal actuation channel and corresponds to the single-chamber driving mode used in the bending tests. Material damage and fracture were not included in the analysis. The resulting deformed configurations and displacement-magnitude contours under the four pressure levels are shown in Figure 3.

The maximum displacement magnitude and maximum von Mises stress obtained at each pressure level are summarized in Table 3.

**Figure 4:**
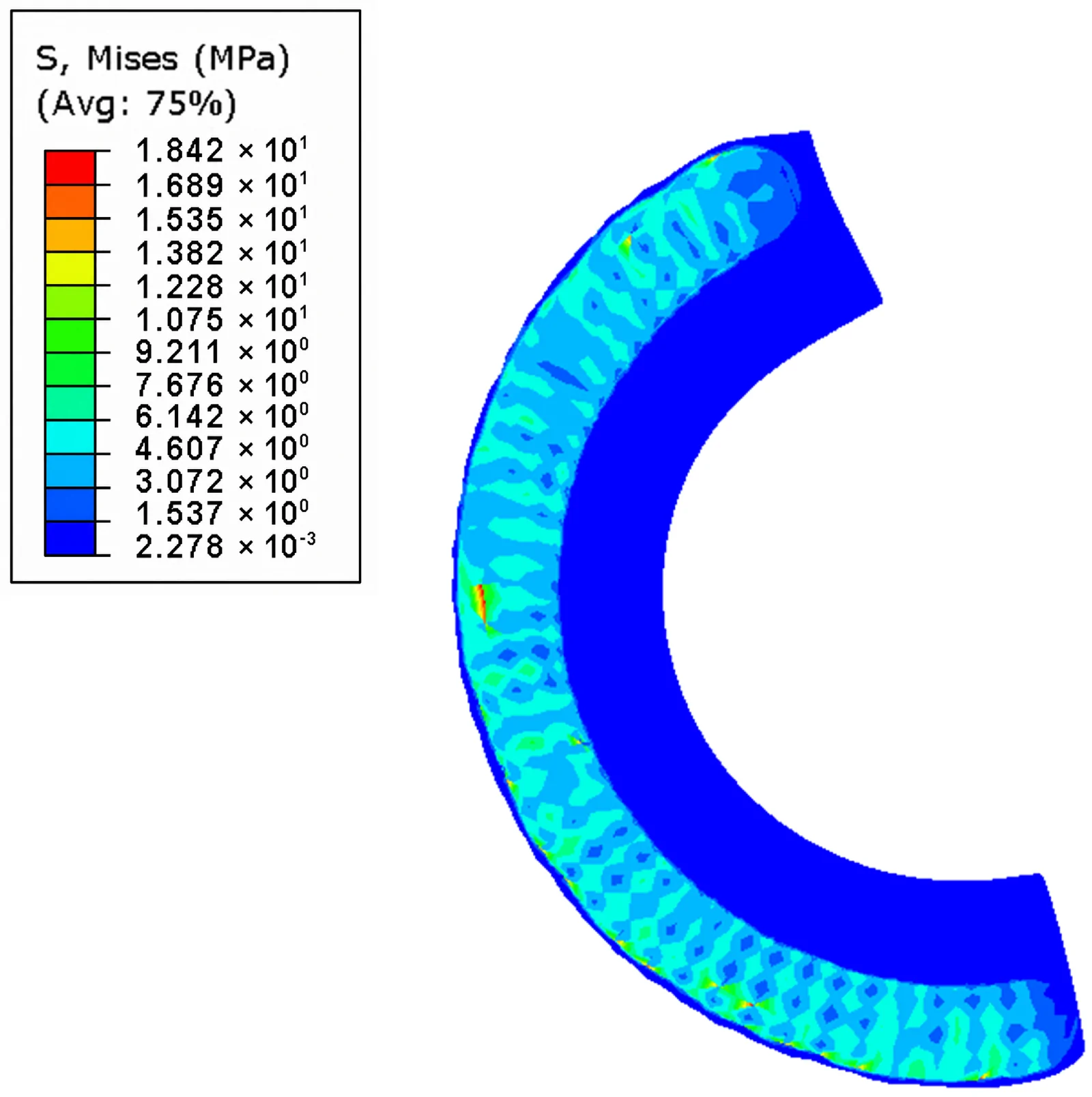
Longitudinal sectional von Mises stress distribution of the proximal segment at 200 kPa. The sectional view shows the stress distribution around the pressurized chamber.

To further examine the internal stress distribution under the maximum applied pressure, a longitudinal sectional view of the proximal segment at 200 kPa was analyzed, as shown in Figure 4.

**Figure 5:**
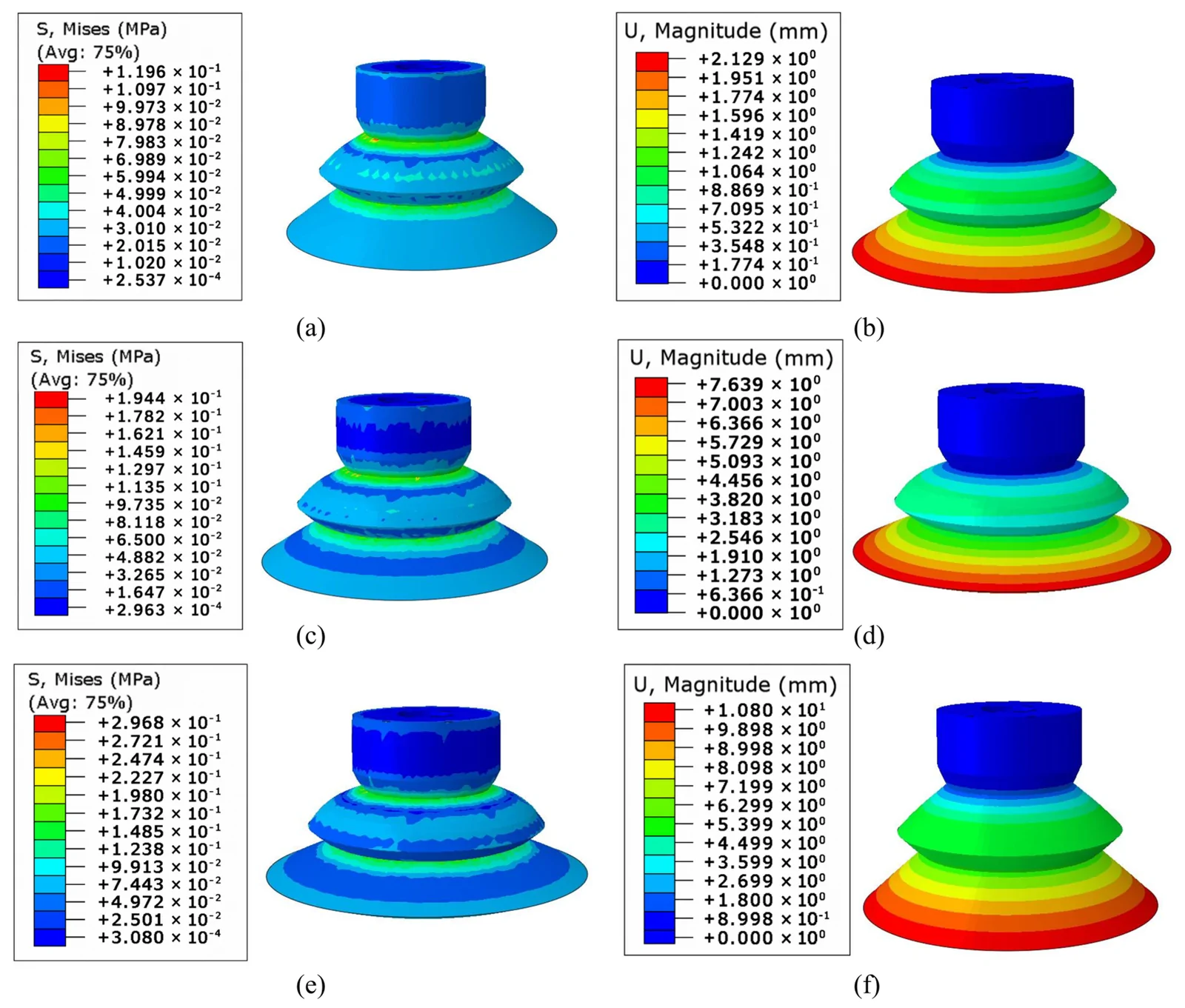
Finite element simulation of the bio-inspired suction cup under different equivalent sealing loads after mechanical compression: (**a**) stress at 10 kPa; (**b**) displacement at 10 kPa; (**c**) stress at 25 kPa; (**d**) displacement at 25 kPa; (**e**) stress at 50 kPa; (**f**) displacement at 50 kPa. The loads are numerical parameters used to examine structural deformation and do not represent measured cavity pressures or predicted suction forces.

The suction-cup deformation was also analyzed using finite element simulation. During mechanical pressing against a target, partial air expulsion and elastic recovery of the cup lip can promote local sealing. Equivalent pressure differences of 10, 25, and 50 kPa were applied to the inner cavity surfaces as parametric post-compression sealing conditions. All three cases used the same cup geometry and material parameters, allowing the structural response to the prescribed equivalent pressure differences to be compared directly. The corresponding von Mises stress and displacement-magnitude fields are presented in Figure 5. The model does not include a target surface, friction, explicit lip separation, leakage, or fluid–structure interaction. Across the three loading cases, deformation was concentrated in the compliant lip and transition regions, whereas the main body retained its overall geometry. Local stress maxima occurred near the geometric transitions between the lip and cup body. As the equivalent pressure difference increased, the same transition region remained the principal location of stress concentration, while the deformation pattern of the cup lip remained smooth. This response is consistent with the intended structural role of the cup: the compliant lip accommodates local geometric adjustment during pressing, whereas the thicker cup body limits excessive collapse of the cavity. The numerical fields were therefore used to compare structural responses at increasing loads and to identify regions that warrant attention in subsequent profile refinement. They do not establish a material-failure threshold, because the model does not include damage initiation or a target-contact interface. The analysis therefore illustrates the structural response of the cup after mechanical pressing; it does not provide pull-off force, retention time, or actual cavity pressure. Together, these results provide a structural context for the cup geometry and material selection used in the prototype. The subsequent fabrication procedure implements this compliant TPU cup and silicone-link arrangement at the distal end of the manipulator.

### 2.4. Fabrication

**Figure 6:**
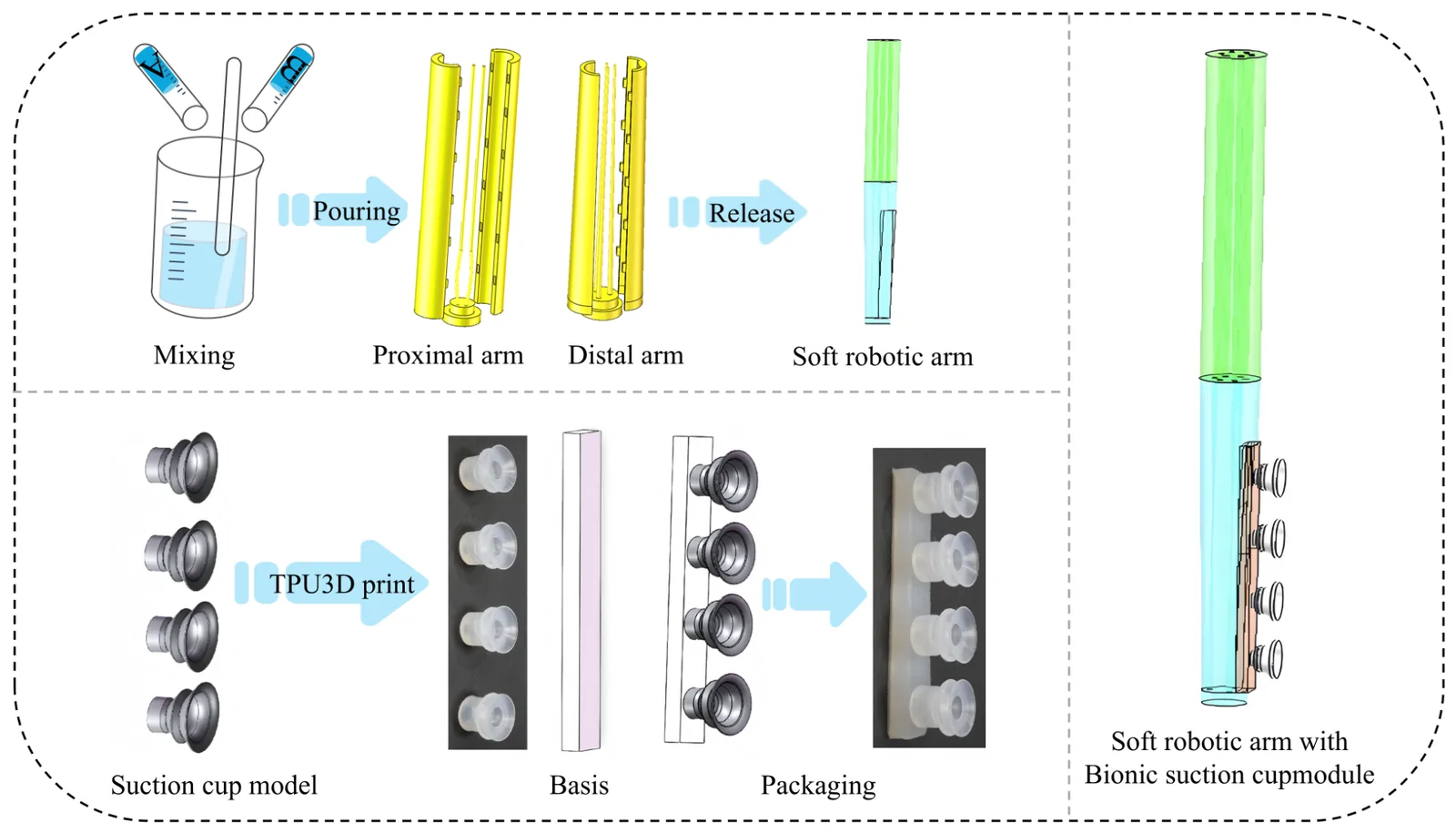
Mold design and casting process for the fabrication of the pneumatic soft manipulator.

The silicone arm and the TPU suction cups were fabricated by different processes because they perform different mechanical functions (Figure 6). The arm body was fabricated by mold casting. External molds and internal cores were first produced by 3D printing according to the designed chamber geometry. Two-component liquid silicone rubber was then mixed, degassed under vacuum, poured into the mold, and cured at room temperature. After demolding, the arm contained the required internal pneumatic chambers. Separate pneumatic paths were retained for the proximal and distal actuation chambers during assembly, thereby preserving independent segment actuation. The suction cups were fabricated from TPU by 3D printing. The printed cups used 85A polyether TPU. The cup wall thickness was 2.5 mm. TPU was selected because it can maintain the cup profile while retaining sufficient flexibility at the lip for contact sealing. Silicone rubber was used for the flexible links between adjacent cups. The compliant links attached the cup array without introducing a rigid connection at the distal end. The cup array and the links were bonded to the distal segment using room-temperature-vulcanizing silicone adhesive. The silicone pneumatic tubes were also bonded with silicone adhesive at the connection regions. No quantitative leakage-rate or long-term sealing test was performed. The cups were not connected to an active vacuum source and relied on mechanically assisted passive sealing during contact. This assembly keeps the distal end compliant and avoids routing additional vacuum tubes through the arm. Compared with sensorized or actively vacuum-driven octopus-inspired grippers, the present prototype emphasizes a simple structural implementation: the pneumatic arm provides reaching and bending, while the distal cups assist local contact after mechanical pressing. This approach is suitable for early-stage prototype validation, although active suction systems would provide better control of attachment pressure and release timing [31].

## 3. Kinematic Model and Workspace Analysis

### 3.1. Piecewise Constant Curvature Model

**Figure 7:**
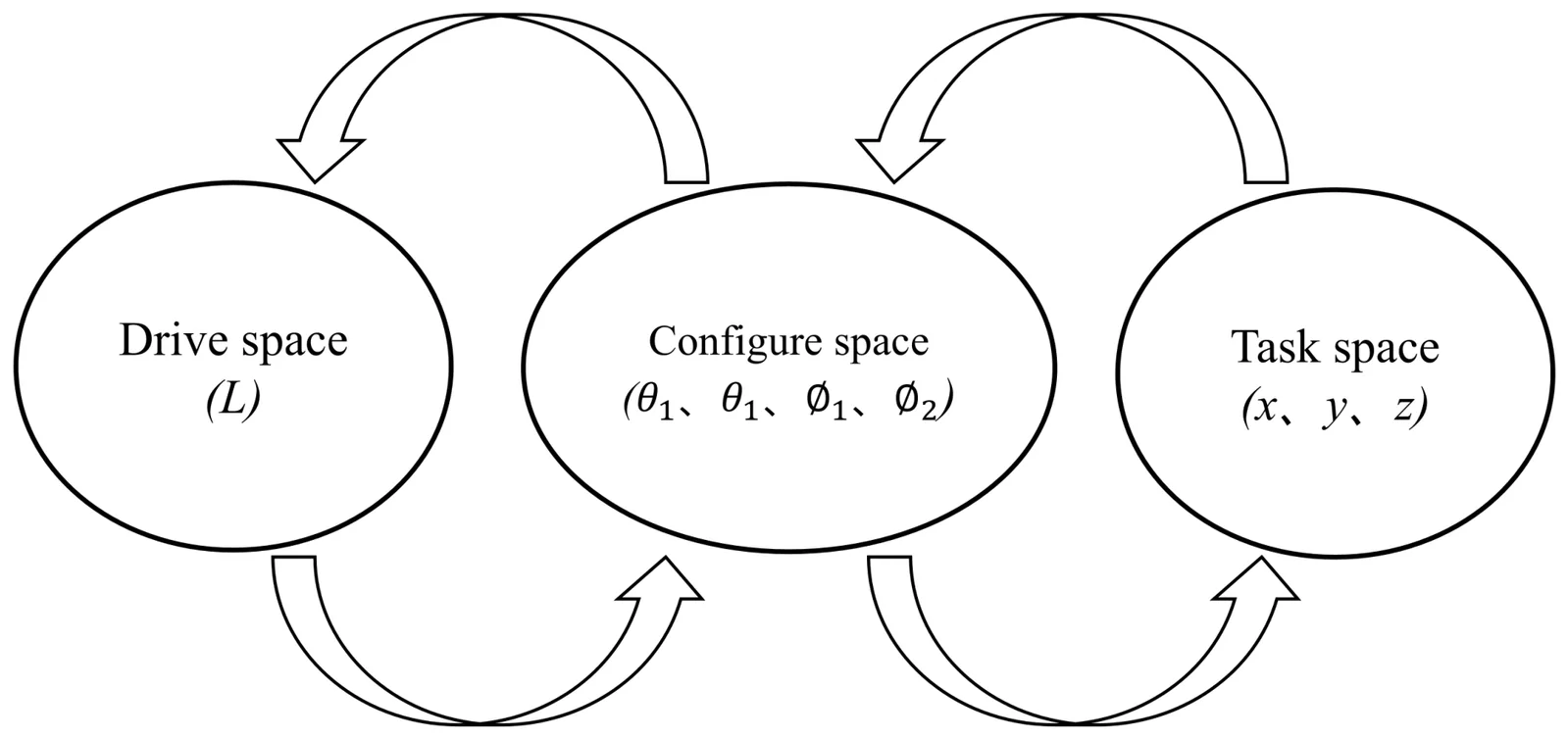
Kinematic mappings between the configuration space and task space of the two-segment soft manipulator based on the piecewise constant curvature assumption. The forward arrow indicates the configuration-to-task mapping, whereas the reverse arrow indicates the corresponding conceptual inverse relation.

Because the manipulator has no discrete revolute joints, its posture was described with a piecewise constant curvature model. The PCC model provides a first-order geometric representation of the two segments. Each segment is represented by an arc with a prescribed bending angle and bending-plane angle, and the distal-end pose is obtained from the two segment transformations (Figure 7). The model describes the geometric relation between segment configuration and end pose; it does not establish a pressure–angle constitutive relation.

**Figure 8:**
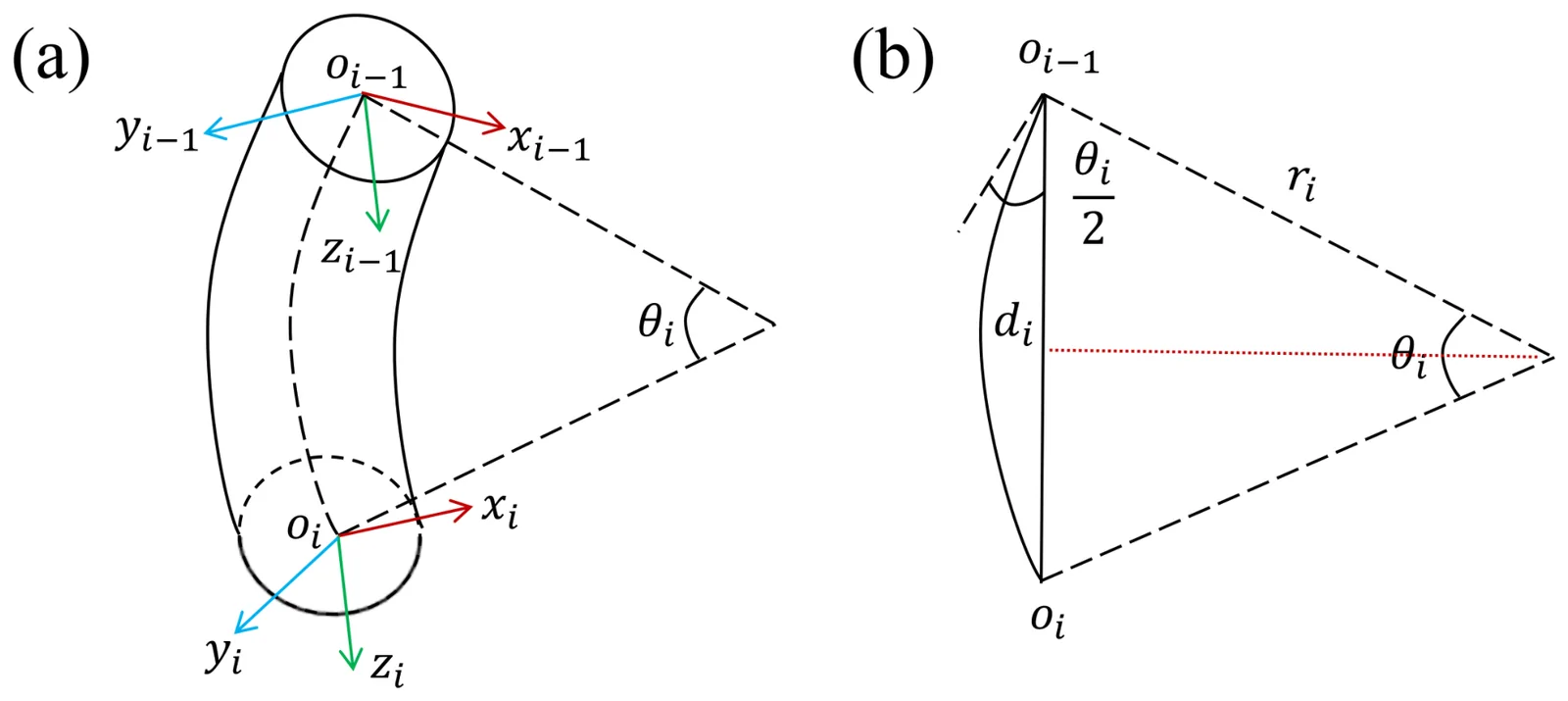
Geometric representation of the soft manipulator under the piecewise constant curvature assumption: (**a**) simplified side-view model; (**b**) geometric representation of the soft arm.

A local coordinate system is defined at the base of each segment, as shown in Figure 8a, and the corresponding circular-arc geometry is illustrated in Figure 8b. The bending plane angle is denoted by ϕ. For a segment with curvature κ=θ/L, the end position in the local coordinate system is written as

(1) $$\left\{\begin{array}{c} x=\frac{L}{\theta }\left(1-\cos\theta \right)\cos\phi ,\\ y=\frac{L}{\theta }\left(1-\cos\theta \right)\sin\phi ,\\ z=\frac{L}{\theta }\sin\theta . \end{array}\right.$$

When θ approaches zero, the straight configuration is obtained by the limiting case x=0, y=0, and z=L.

The homogeneous transformation of the *i*th segment can be expressed as

(2) Ti  i−1=T11T12T13T14T21T22T23T24T31T32T33T340001,

where

$$\begin{array}{cc} T_{11} & =\sin^{2}\phi +\cos^{2}\phi \cos\theta ,\\ T_{12}=T_{21} & =\cos\phi \sin\phi (1-\cos\theta ),\\ T_{13} & =\cos\phi \sin\theta ,\\ T_{14} & =L\cos\phi (1-\cos\theta )/\theta ,\\ T_{22} & =\cos^{2}\phi +\sin^{2}\phi \cos\theta ,\\ T_{23} & =-\sin\phi \sin\theta ,\\ T_{24} & =L\sin\phi (1-\cos\theta )/\theta ,\\ T_{31} & =-\cos\phi \sin\theta ,\\ T_{32} & =\sin\phi \sin\theta ,\\ T_{33} & =\cos\theta ,\\ T_{34} & =L\sin\theta /\theta . \end{array}$$

For the two-segment arm, the end pose is obtained by multiplying the transformation matrices of the proximal and distal segments:

(3) T20=T10T21=RP01=nxoxaxpxnyoyaypynzozazpz0001,

where R is the rotation matrix and $P={\left[p_{x},p_{y},p_{z}\right]}^{T}$ is the end-position vector.

### 3.2. Workspace Analysis

**Figure 9:**
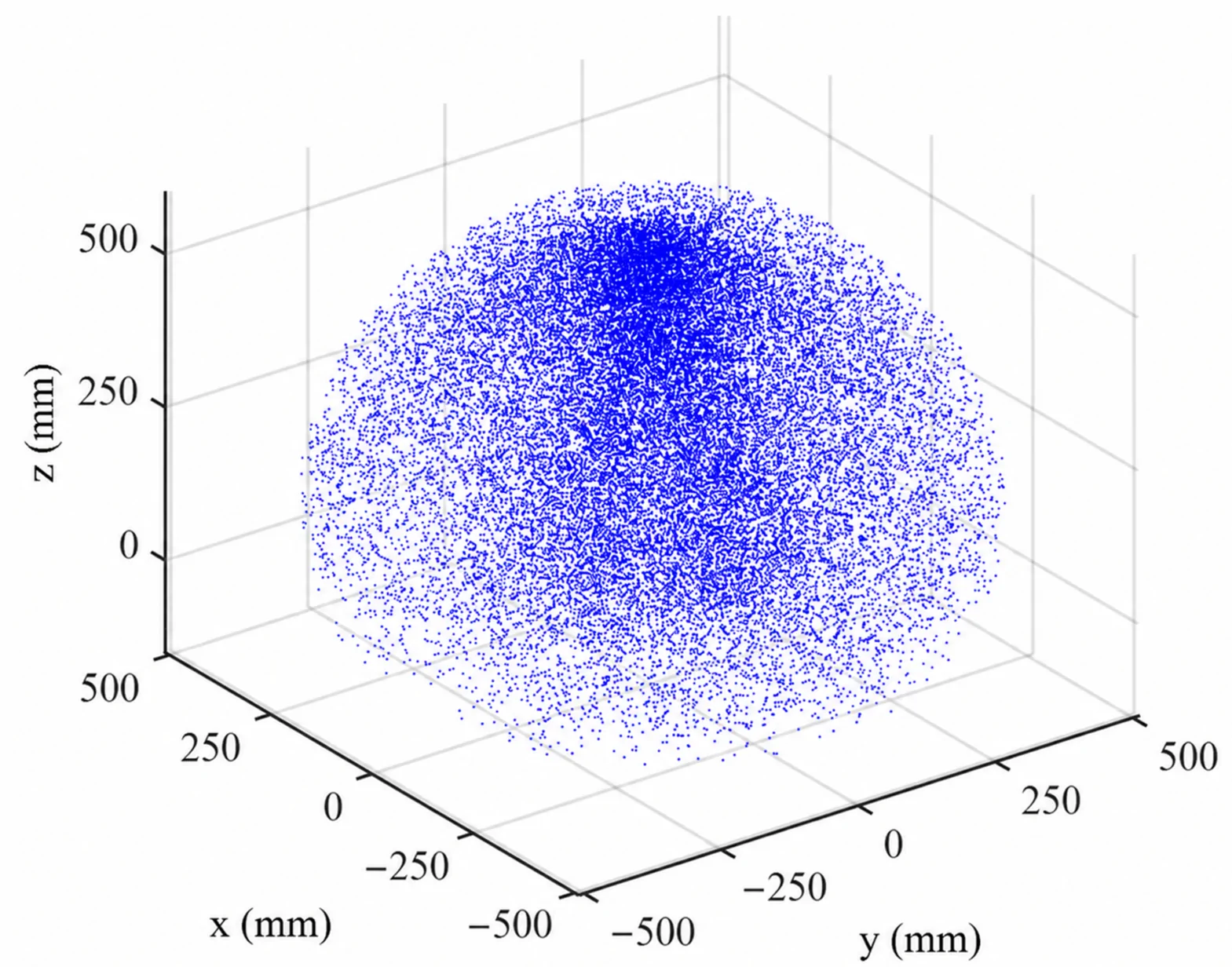
Workspace of the dual-segment pneumatic soft manipulator obtained from kinematic analysis and Monte Carlo simulation. The point cloud is a theoretical geometric envelope and not an experimentally verified reachable workspace of the prototype.

The reachable workspace was estimated by combining the kinematic model with Monte Carlo sampling. The sampled variables were $\theta_{1}\in \left[0,180^{{}^{\circ}}\right]$, $\phi_{1}\in \left[0,360^{{}^{\circ}}\right]$, $\theta_{2}\in \left[0,180^{{}^{\circ}}\right]$, and $\phi_{2}\in \left[0,360^{{}^{\circ}}\right]$. For each sampled configuration, the distal-end position was calculated from Equation (3). The point cloud in Figure 9 gives the theoretical reachable region. The 0°–180° bounds specify the geometric sampling range used for workspace visualization. The distribution also shows why the two-segment layout was used: the proximal segment changes the approach direction, while the distal segment adjusts the local contact posture near the object. The model is used here as a first-order design tool. It does not include hysteresis, viscoelastic relaxation, pressure-delay effects, chamber-wall nonlinearity, or deformation caused by payload. These effects are common in pneumatic soft actuators and become more important during fast motion, long holding periods, or manipulation of heavier objects. For this reason, the workspace should be interpreted as a theoretical reachable region rather than a guaranteed manipulation envelope.

## 4. Experimental Validation

### 4.1. Experimental Platform

**Figure 10:**
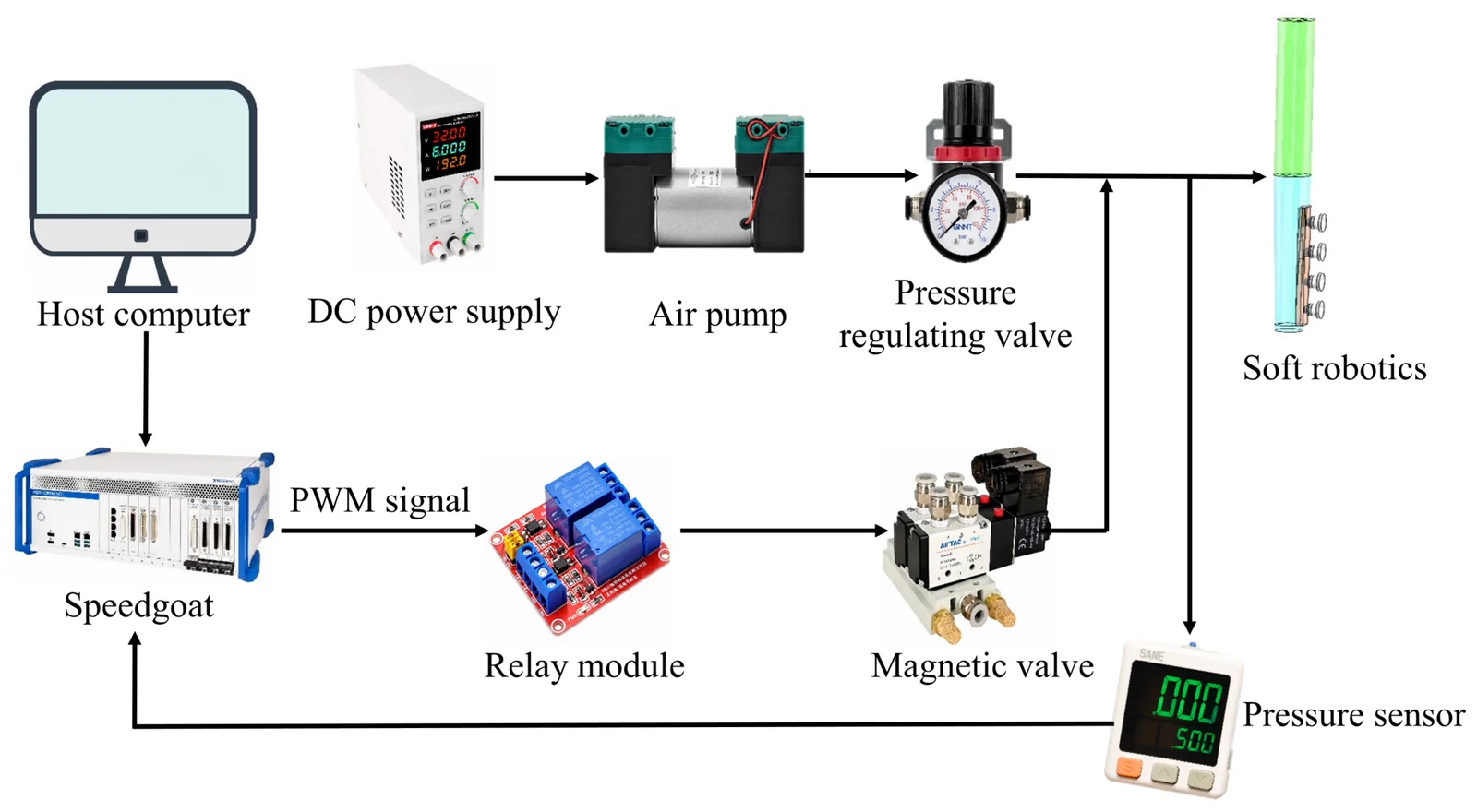
Experimental platform for the soft manipulator. The Speedgoat unit is used as the controller. The AR2000 unit is a pressure-regulating valve with a mechanical pressure gauge; no independent electronic pressure sensor or closed-loop pressure feedback is used in the present platform.

A pneumatic test platform was constructed for pressure regulation, bending measurement, and object-contact tests (Figure 10). The platform included a pneumatic source, an AR2000 pressure-regulating valve with a mechanical pressure gauge, H103-EL solenoid valves, a Speedgoat real-time controller, and a host computer. The Speedgoat controller generated the control commands for solenoid-valve actuation. Pressure was set and read using the AR2000 mechanical gauge; the platform did not use an independent electronic pressure sensor or closed-loop pressure feedback. The valves supplied the proximal and distal chambers through separate pneumatic paths. The distal suction cups were tested by pressing them against target objects using the soft manipulator; no independent vacuum source was used. The bending angle was read using a 360° protractor placed behind the arm as a visual reference for approximately steady configurations.

### 4.2. Bending and Manipulation Tests

**Figure 11:**
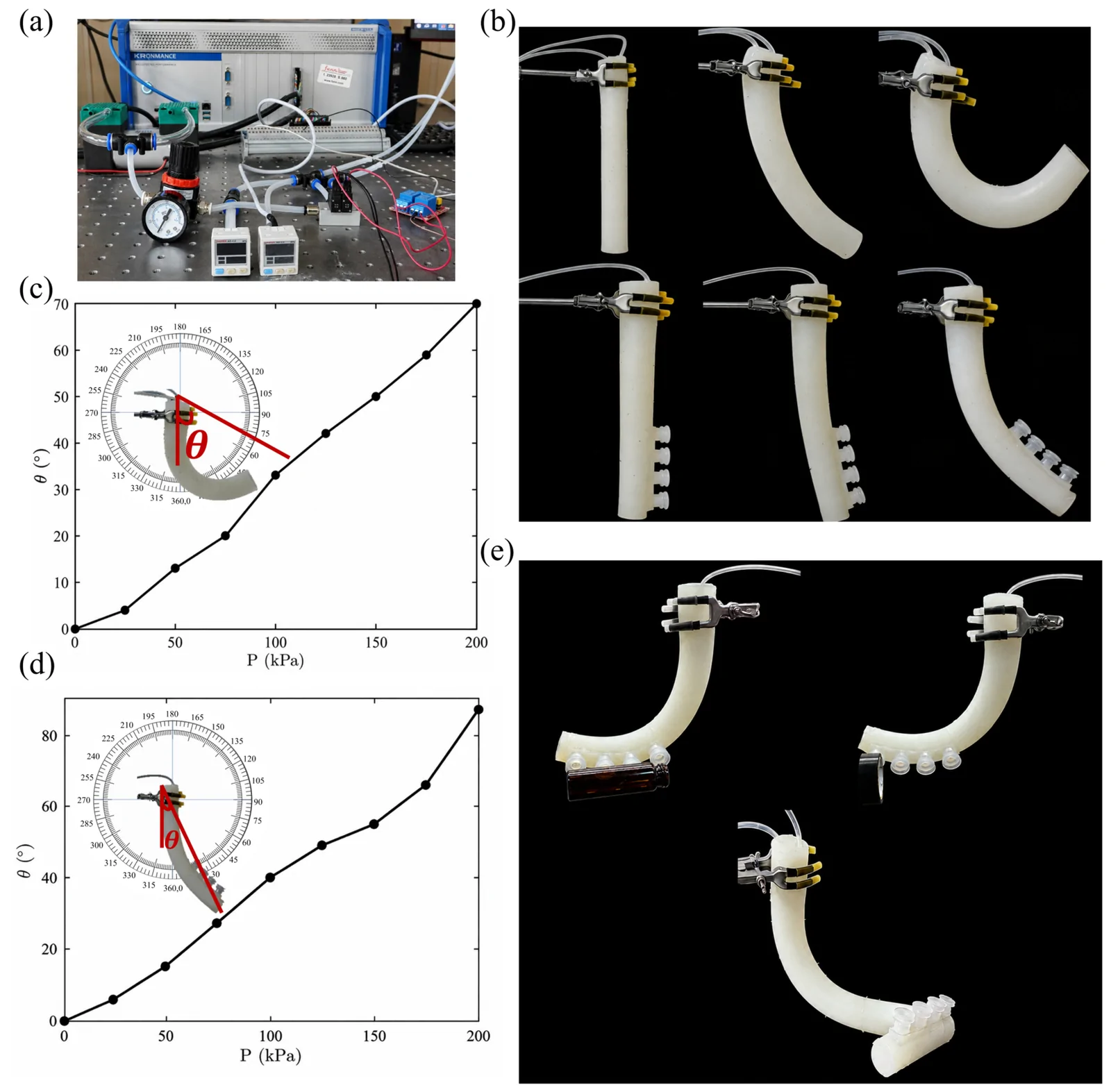
Experimental setup and validation results: (**a**) pneumatic control hardware and test platform; (**b**) bending configurations of the proximal and distal pneumatic segments; (**c**) pressure–bending angle response of the proximal segment; (**d**) pressure–bending angle response of the distal segment; (**e**) passive suction-cup-assisted distal contact with a brown glass bottle and a black roll of electrical tape (**upper**), and compound bending and twisting motion generated by coordinated proximal–distal actuation (**lower**).

The experimental setup used for bending and manipulation tests is shown in Figure 11a. Single-segment bending tests were first conducted to evaluate the pressure response of the proximal and distal modules. Step pressures from 50 to 200 kPa were applied to a single chamber to induce unidirectional bending, while the remaining chambers were unpressurized. At each pressure level, the segment was allowed to reach a steady configuration before the bending angle was recorded from the 360° protractor. As shown in Figure 11b–d, both segments showed increasing bending angles with increasing pressure, indicating controllable pneumatic deformation within the tested range. At 200 kPa, the maximum observed bending angles were approximately 70° for the proximal segment and 87° for the distal segment. The available records do not provide a complete repeatability dataset; repeatability is therefore not quantified here. The distal segment showed a larger pressure response than the proximal segment, which is consistent with its smaller cross-section and lower bending stiffness. This result supports the intended functional division between the two segments: the proximal segment provides structural support and global positioning, while the distal segment provides more compliant local motion near the contact region. Two-segment motion was then tested by applying different pressure combinations to the proximal and distal chambers. Coordinated actuation allowed the arm to form compound postures, including bending and twisting configurations. These tests show that the two segments do not only work as independent bending modules but can also cooperate to adjust the overall spatial posture of the arm. Figure 11e shows distal-segment object-contact tests with a brown glass bottle and a black roll of electrical tape. The glass bottle had a mass of 25.62 g, a diameter of 27.6 mm, and a height of 75.2 mm. The tape roll had a mass of 59.12 g, an outer diameter of 68.2 mm, and a thickness of 18.4 mm. In these demonstrations, the cups were mechanically pressed against the target surface before holding the object. The lower portion of Figure 11e shows combined bending of the proximal and distal segments, demonstrating the ability of the two-segment structure to generate spatial deformation. The object-contact tests are preliminary demonstrations of the distal suction-cup interface rather than a full characterization of adhesion performance. No controlled holding-duration, pull-off-force, payload, surface-curvature, or repeated-cycle test was performed; the demonstrations are therefore qualitative evidence of passive distal contact only. The holding behavior of the cups may vary with surface condition, curvature, pressing force, sealing quality, and cup-lip deformation. Future work should quantify holding force, payload, repeatability, fatigue, and failure modes under controlled test conditions.

## 5. Conclusions

This study presented an octopus-inspired modular two-segment pneumatic soft manipulator with passive suction cups at the distal end. The design follows the functional division observed in octopus arms, where the compliant body provides reaching and bending, and the suckers assist local contact after the arm reaches the target. In the proposed manipulator, the cylindrical proximal segment mainly provides structural support and global bending, while the tapered distal segment improves local compliance and contact posture adjustment. The two pneumatic segments can be actuated separately, allowing the manipulator to form compound spatial postures through coordinated pressurization. Finite element analysis was used to examine the pressure-driven deformation of the arm and the structural response of the TPU suction cups. A piecewise constant curvature model was established to estimate the posture and reachable workspace of the two-segment structure. The workspace provides a theoretical geometric envelope for the two-segment configuration. Prototype experiments further verified the pressure response of the proximal and distal segments. Within the tested range of 50–200 kPa, the bending angle increased with input pressure, and the maximum observed bending angles at 200 kPa were approximately 70° for the proximal segment and 87° for the distal segment. The distal cups made local contact with a brown glass bottle and a black roll of electrical tape in qualitative demonstrations. These observations show the feasibility of passive distal contact but do not quantify holding force, payload, retention time, or repeatability. Coordinated actuation of the proximal and distal segments also produced bending and twisting configurations, confirming the feasibility of the modular two-segment architecture for spatial soft manipulation. The passive suction cups provide a simple distal contact interface, and their holding behavior depends on surface condition, curvature, pressing force, and cup-lip deformation. Future work will quantify suction force, payload, attachment cycles, fatigue, and pressure-feedback control. Potential application scenarios include inspection and light-object handling in confined or irregular environments, where compliant posture adjustment and local surface conformity are desirable.

## Figures and Tables

**Table 1:** Comparison of the present bio-inspired soft manipulator with closely related octopus-inspired systems.

Reference	Arm Architecture and Actuation	Distal Interface	Distinguishing Design Feature
Mazzolai et al. [28]	Octopus-inspired soft arm	Suction cups	Integration of a compliant arm and suction cups for grasping in confined environments.
Xie et al. [29]	Tapered pneumatic soft actuator	Suckers	Tapered geometry and sucker-assisted grasping.
Wu et al. [31]	Dual-segment, multi-wire-driven soft arm	Integrated suction cups	Cable-driven dual-segment actuation with integrated cups.
This work	Cylindrical proximal segment and tapered distal segment; each has three pneumatic chambers at 120°	Passive, vacuum-free TPU cup array	Separate pneumatic actuation of the proximal and distal segments for global posture adjustment and local contact adjustment.

**Table 2:** Finite-element settings and material parameters.

Item	Setting
Software and analysis	Abaqus; static large-deformation analysis; mm–N–s–MPa unit system.
Arm discretization	C3D10H quadratic hybrid tetrahedra; global seed 6 mm; 24,619 nodes; 15,889 elements.
Arm boundary and load	Proximal end fully fixed; one proximal actuation-chamber inner wall pressurized at 50, 100, 150, or 200 kPa; the other two proximal actuation chambers at zero gauge pressure.
Silicone model	Third-order Yeoh model: $C_{10}=0.090036$ MPa, $C_{20}=-0.0038806$ MPa, $C_{30}=0.001524$ MPa, $D_{1}=0.1111$ MPa^−1^, $D_{2}=D_{3}=0$, ρ=1072 kg m^−3^ [33,34].
Cup model	Third-order Yeoh model: $C_{10}=4.8$ MPa, $C_{20}=-0.52$ MPa, $C_{30}=0.075$ MPa, $D_{1}=0.002$ MPa^−1^, $D_{2}=D_{3}=0$, ρ=1140 kg m^−3^. The TPU properties were represented by a literature-informed parameter set for comparable 85A polyether TPU [35].
Cup loading scope	Equivalent pressure differences of 10, 25, and 50 kPa applied to the inner cavity surfaces as parametric post-compression sealing conditions.

**Table 3:** Summary of the maximum displacement magnitude and maximum von Mises stress obtained from the arm finite-element model.

Pressure (kPa)	Maximum Displacement (mm)	Maximum von Mises Stress (MPa)
50	198	8.60
100	229	10.07
150	271	15.51
200	302	18.42

## Data Availability

The data presented in this study are available upon request from the corresponding author.
